# Age at first birth and long-term mortality for mothers: the Ohsaki cohort study

**DOI:** 10.1186/s12199-017-0631-x

**Published:** 2017-04-04

**Authors:** Taichi Sakai, Yumi Sugawara, Ikue Watanabe, Takashi Watanabe, Yasutake Tomata, Naoki Nakaya, Ichiro Tsuji

**Affiliations:** 1 Juntendo University Faculty of Health Sciences and Nursing, 3-7-33 Omiyacho, Mishima, Shizuoka 411-8787 Japan; 2 Division of Epidemiology, Department of Health Informatics and Public Health, Tohoku University School of Public Health, Graduate School of Medicine, Sendai, Japan; 3 Department of Nursing, Faculty of Health Science, Tohoku Fukushi University, Sendai, Japan; 4 Department of Preventive Medicine and Epidemiology, Tohoku Medical Megabank Organization, Tohoku University, Sendai, Japan

**Keywords:** Age at first birth, Cohort study, Japan, Mortality

## Abstract

**Background:**

Although maternal age at first birth has been rising in many developed countries, its long-term effects on the health of the mothers themselves are unclear. In this study, we investigated the relationship between maternal age at first birth and long-term mortality.

**Methods:**

We conducted a cohort study of 20,624 parous Japanese women aged between 40 and 79 years in 1994 and followed up their survival for 14 years. Based on maternal age at first birth, the women were divided into four groups: ≤19 years, 20–24 years, 25–29 years, and ≥30 years. Using the 20–24 years group as a reference, hazards ratios (HRs) for all-cause and cause-specific mortality were calculated.

**Results:**

Multivariate HRs for all-cause mortality were 1.17 in the ≤19 years group, 1.09 in the 25–29 years group, and 1.33 in the ≥30 years group. A U-shaped relationship was apparent between maternal age at first birth and mortality. This relationship was also observed for mortality attributable to cancer, cardiovascular disease and other diseases. This U-shaped relationship was observed only for women born before 1935 and the birth year of the first child before 1960.

**Conclusion:**

A U-shaped relationship was apparent between maternal age at first birth and mortality. As maternal age at first birth is rising worldwide, the risk it imposes appears to have significance in the context of public health.

## Background

The age at which a woman has her first child (“maternal age at first birth”) has been rising in many countries. The highest countries of the average age at first birth among the Organisation for Economic Co-operation and Development (OECD) member countries were Japan (30.3 years), Switzerland (30.3 years), and Spain (30.3 years); conversely, the lowest country was Estonia (26.5 years) in 2012 [1]. In Japan, the mean maternal age at first birth was 25.6 years in 1970, rising to 30.3 years in 2012, and is projected to rise further. This trend is a same phenomenon in other countries. In Switzerland, the mean maternal age at first birth was 25.3 years in 1970, rising to 30.3 years in 2012. In Spain, the mean maternal age at first birth was 25.0 years in 1980 (No data in 1970), rising to 30.3 years in 2012. In Estonia, the mean maternal age at first birth was 22.1 years in 1970, rising to 25.6 years in 2012. The rising trend of the mean maternal age at first birth can also be seen in other OECD member countries. A number of studies have reported that higher age at first birth is associated with increased health risks to both mothers and their children. For example, children are thought to have a higher risk of Down syndrome and fetal death [2, 3]. Likewise for the mothers, there is an increased risk of pregnancy-associated toxemia and maternal complications during labor [4–7].

Furthermore, life-long effects of the age at first birth on the mother have been indicated. In particular, associations with cancer and cardiovascular diseases have often been reported. For breast cancer, studies have agreed that the risks of incidence and mortality are significantly elevated when women become first-time mothers after 30 years of age [8–10]. For other cancers, results have been inconsistent. Some studies have indicated that older maternal age at first birth is significantly associated with increased mortality because of ovarian cancer [11] and the risk of incident colon cancer [12]. On the other hand, other studies have found that a younger maternal age at first birth significantly increases the incidence risk of ovarian cancer [13] and decreases that of colon cancer [14]. Studies indicating both increased and decreased risks of late age at first birth have been reported for pancreatic [15, 16] and lung cancers [17, 18], although no association with age at first birth has been demonstrated for stomach cancer [19]. With regard to stroke, a tendency for an increased risk with higher age at first birth has been reported [20], whereas a decreasing trend has been found for coronary heart disease [21].

Some studies have investigated the relationship between all-cause mortality and age at first birth. However, those studies focused mainly on the risk to younger childbearing women. In particular, an increase in all-cause mortality in later life has been reported for women who became first-time mothers before 20 years of age [22–24]. As for the mechanisms involved, a relationship between early childbearing and socio-economic status has been discussed.

Considering that the risk of diseases such as breast cancer is increased in women who are older at the time of their first birth, all-cause mortality may also be increased in these women. In fact, studies targeting African–American women have shown that all-cause mortality increases in those who first become mothers at an older age [25, 26]. This finding suggests that the association between the timing of motherhood and risk of disease may depend on ethnicity. However, no studies of this type targeting Asians have been reported. As age at first birth has been rising in many developed countries, clarifying the health effects of such changes is an important public health issue.

The purpose of this prospective cohort study was to investigate the relationship between maternal age at first birth and long-term mortality in the general population of a local community in Japan. Our intention was to clarify the importance of “maternal age at first birth” as a reproductive factor affecting the life-long health of women. As maternal age at first birth has been rising worldwide, clarification of its effects on life-long health and longevity would be informative in the context of women’s health, reproductive health and rights.

## Methods

### Study cohort

A brief overview of the study design is provided below, as the details of the Ohsaki National Health Insurance (NHI) Cohort Study have been reported elsewhere [27]. NHI beneficiaries (26,481 men and 28,515 women) between 40 and 79 years of age living in the catchment area of the Ohsaki Public Health Center in Miyagi Prefecture were invited to participate in this study. Self-administered questionnaires requesting medical and reproductive histories along with information on health-related lifestyle habits were distributed between October and December 1994. A trained researcher distributed a self-administered questionnaire that included an explanation of the purpose of the study and the participant’s right to withdraw from the study at any time. One week later, the questionnaire was collected by the same researcher. Although we did not ask for written consent, submission of questionnaires was regarded as consent to participate. Among the 28,515 women, 27,134 responded, giving a response rate of 95.2%.

Among these women, we excluded those who had never had children (*n =* 2,674), those who died or withdrew from the NHI before the start of follow-up (*n =* 454), those who reported a history of cancer or cardiovascular disease (stroke and myocardial infarction) at the time of baseline data collection (*n =* 1,998), and those who did not record their age at first birth (*n =* 1,384). As a result, 20,624 women (76.0%) formed the study cohort and were followed up for 14 years from January 1, 1995 to December 31, 2008 to determine their vital status (dead or alive). For women who died during this period, the date and cause of death were recorded.

All study protocols were approved by the Ethics Committee of Tohoku University School of Medicine.

### Exposure data

Items in the self-administered questionnaire included reproductive history, age at first birth, number of pregnancies, number of births, and use of oral contraceptives. To determine age at first birth, participants provided a written answer to the question “How old were you when you first gave birth (including stillbirths)?” However, women’s ages at which they had a miscarriage or abortion at first birth were unavailable because we did not include relevant questions in our questionnaire.

### Follow-up

The end point was mortality (all-cause and cause-specific mortality). Death of a participant and withdrawal from the NHI system were confirmed using the Ohsaki NHI database. Cause of death was confirmed by reference to the Vital Statistics Records (Death Certificate Notification). Since vital information on the participants who withdrew from the NHI system was no longer available, they were censored at the date of withdrawal.

Causes of death in the Vital Statistics Record (Death Certificate Notification) were assessed and classified by a trained researcher based on the International Classification of Diseases, version 10 (ICD-10). The ICD-10 codes for death caused by cardiovascular diseases and cancer are I00-I99 and C00-C97, respectively. During the 14 years of follow-up, we confirmed the death of 2,165 participants.

### Statistical analysis

We counted person-years of observation from January 1, 1995 to the date of death, withdrawal from the NHI, or December 31, 2008, whichever came first. The total number of person-years of observation was 232,921. We analyzed the data which excluded participants who died after 1 year follow-up, but because there was not much difference between the results with all data and the results with excluded data, we decided to use all data (Table 5 in Appendix).

In accordance with previous studies [8, 13, 18, 19], participants were divided into four groups based on maternal age at first birth: ≤19 years, 20–24 years, 25–29 years, and ≥30 years. A Cox proportional hazards model was used to calculate hazard ratios (HRs) and 95% confidence intervals (CIs) for mortality (all-cause and cause-specific mortality) using the 20–24-year group as a reference. In addition, stratified analysis based on age at baseline (<60 years vs. ≥60 years) and the birth year of the first child (<1960 vs. ≥1960) was undertaken to determine the association between age at first birth and mortality. Because the participants, whose baseline age was 60 years, were born in 1935, the cutoff of the birth year of their first children was defined as 1960 that mother’s mean age at first birth, 25 years old, was added on the year 1935.

The following variables were considered as covariates: age at baseline (continuous variate), number of births (1, 2, 3, ≥4), use of oral contraceptives (yes or no), consumption of green vegetables, carrot or pumpkin, orange (for each food, ≤1–2 times per month, 1–2 times per week, 3–4 times per week, or almost every day), consumption of coffee (<1, 1–2, 3–4, or ≥5 cups per day), smoking (never smoker, former smoker, current smoker), alcohol consumption (never drinker, former drinker, current drinker), body mass index (BMI; <18.5, 18.5–24.9, and ≥25.0), increase in body weight since age 20 years (<10.0 or ≥10.0 kg), walking duration (<1 or ≥1 h per day), sleeping time in hours per day (continuous variable), defecation frequency (≥1 time per day, 1 time per 2–3 days, 1 time per 4–5 days or ≤1 time per 6 days), years of education (<10 years, 10–12 years, ≥13 years), ikigai (yes, uncertain, or no), perceived mental stress (high, moderate, or low), and history of diabetes (yes or no). BMI was calculated by dividing body weight at the baseline by height (in meters) squared.

All tests were two-sided and differences at *p* < .05 were considered statistically significant. SAS version 9.3 software (SAS Institute, USA) was used for all statistical analyses.

## Results

Table 1 displays the basic characteristics of the participants grouped by maternal age at first birth. A positive correlation was evident between age at first birth and years of education. The proportion of the participants with university or higher degrees in the ≤19 years group was 1.6% compared to 16.0% in the ≥30 years group. While the average number of births over the lifetime of the mother was 3.9 in the ≤19 years group, the corresponding figure was 2.0 in the ≥30 years group. An inverse correlation between maternal age at first birth and the number of births existed. The proportion of current smokers was 18.9% in the ≤19 years group, 7.4% in the 20–24 years group, 7.0% in the 25–29 years group, and 11.9% in the ≥30 years group. These figures indicated that a large number of current smokers in the ≤19 years and ≥30 years groups existed. No differences were seen in baseline age and green tea consumption.

**Table 1:** Characteristics of Age at First Birth, the Ohsaki Cohort, Japan, 1995–2008

	Age at first birth (years)				
	≤19	20–24	25–29	≥30	*p*-value
No. of subjects	498	13,607	5,625	894	
Age at baseline (%)					
40–49	19.7	18.2	15.4	21.4	<0.01
50–59	20.5	26.1	24.4	18.9	
≥ 60	59.8	55.7	60.2	59.7	
Average (*SD*)	61.3 (11.4)	60.0 (9.9)	60.7 (9.6)	60.3 (10.4)	<0.01
Number of births (%)					
1	2.8	3.2	8.1	34.8	<0.01
2	20.5	33.6	44.2	43.5	
3	28.3	36.5	31.1	15.2	
≥ 4	48.4	26.8	16.6	6.6	
Average (*SD*)	3.9 (1.9)	3.1 (1.3)	2.6 (1.1)	2.0 (1.0)	<0.01
Use of oral contraceptives (%)					
No	95.0	94.8	96.2	97.0	<0.01
Yes	5.0	5.2	3.8	3.0	
Green vegetables consumption (%)					
≤ 1–2 times per month	11.1	7.5	7.1	7.0	<0.01
1–2 times per week	29.2	25.5	25.9	29.1	
3–4 times per week	36.3	36.5	35.8	32.1	
Almost every day	23.5	30.5	31.2	31.8	
Carrot or pumpkin consumption (%)					
≤ 1–2 times per month	17.6	10.6	9.7	10.6	<0.01
1–2 times per week	32.8	32.0	29.9	33.7	
3–4 times per week	32.6	36.9	38.2	32.5	
Almost every day	17.1	20.4	22.2	23.2	
Orange consumption (%)					
≤ 1–2 times per month	11.6	8.2	8.9	10.8	<0.01
1–2 times per week	18.5	16.0	16.5	17.7	
3–4 times per week	30.8	30.4	30.0	29.5	
Almost every day	39.1	45.4	44.6	42.1	
Green tea consumption (%)					
< 1 cup per day	24.2	22.4	21.7	24.9	0.27
1–2 cups per day	18.0	20.8	20.6	20.6	
3–4 cups per day	20.1	23.1	23.6	22.6	
≥ 5 cups per day	37.8	33.6	34.1	32.0	
Coffee consumption (%)					
< 1 cup per day	63.9	58.8	60.4	57.0	<0.01
1–2 cups per day	25.3	31.9	30.5	30.8	
3–4 cups per day	5.1	6.7	6.3	7.8	
≥ 5 cups per day	5.6	2.6	2.7	4.4	
Smoking status (%)					
Current	18.9	7.4	7.0	11.9	<0.01
Former	2.0	1.9	2.6	4.3	
Never	79.0	90.8	90.4	83.9	
Alcohol consumption (%)					
Current	29.3	27.8	22.8	23.7	<0.01
Former	7.7	4.6	4.1	7.8	
Never	63.1	67.6	73.1	68.5	
BMI at baseline in kg/m^2^ (%)					
< 18.5	3.8	3.2	4.4	4.7	<0.01
18.5–24	58.1	63.6	65.5	65.2	
≥ 25.0	38.0	33.2	30.1	30.1	
Average (*SD*)	24.1 (3.4)	23.9 (3.4)	23.6 (3.4)	23.5 (3.7)	<0.01
Increase in body weight since age 20 years (%)					
< 10.0 kg	70.5	76.1	77.8	74.3	<0.01
≥ 10.0 kg	29.6	23.9	22.2	25.7	
Average (*SD*)	4.2 (11.1)	3.5 (9.5)	2.9 (9.6)	3.7 (9.9)	<0.01
Walking duration (%)					
< 1 h per day	42.9	46.5	38.3	34.6	<0.01
≥ 1 h per day	57.1	53.5	61.7	65.4	
Sleeping time (hours per day)					
Average (*SD*)	7.6 (1.4)	7.5 (1.2)	7.4 (1.2)	7.2 (1.2)	<0.01
Defecation frequency (%)					
≥ 1 time per day	70.7	74.0	73.6	70.3	<0.01
1 time per 2–3 days	24.4	23.7	24.2	26.0	
1 time per 4–5 days	3.8	2.0	2.0	3.1	
≤ 1time per 6 days	1.1	0.3	0.3	0.6	
Educational level (%)					
Junior high school (<10 years)	83.5	57.5	52.0	42.6	<0.01
High school (10–12 years)	14.9	36.3	36.2	41.5	
University or higher (≥13 years)	1.6	6.2	11.8	16.0	
Ikigai (%)					
Yes	53.5	58.1	57.8	55.8	<0.01
Uncertain	38.8	37.8	37.7	39.6	
No	7.7	4.1	4.6	4.6	
Perceived mental stress (%)					
High	17.2	17.6	17.6	23.2	<0.01
Moderate	64.1	66.1	66.3	58.3	
Low	18.8	16.3	16.1	18.5	
History of hypertension (%)					
Yes	27.5	27.6	28.3	25.8	0.47
No	72.5	72.5	71.8	74.2	
History of diabetes (%)					
Yes	8.8	5.0	5.3	5.3	<0.01
No	91.2	95.0	94.7	94.7	

Note. *BMI* body mass index, *SD* standard deviation

*P*-value calculated by analysis of variance or χ^2^ test

A U-shaped relationship was apparent between age at first birth and all-cause mortality (Table 2). When using the 20–24 years group as a reference, the multivariate-adjusted HR was significantly increased to 1.33 (95% CI, 1.08–1.62; *p* < .01) in the ≥30 years group. Although not statistically significant, a tendency toward increased mortality because of stroke and cerebral infarction were indicated in the ≤19 years group.

**Table 2:** Cox Proportional Hazard Ratios (HRs) of Mortality Because of Cardiovascular Disease and Cancer According to Age at First Birth, the Ohsaki Cohort, Japan, 1995–2008

	Age at first birth (years)			
	≤19	20–24	25–29	≥30
	(*n* = 498)	(*n* = 13,607)	(*n* = 5,625)	(*n* = 894)
Person-years	5,347	153,247	64,446	9,881
All-cause				
No. of deaths	76	1352	617	120
Crude HR (95% CI)	1.62 (1.29–2.04)	1.00 (referent)	1.08 (0.99–1.19)	1.38 (1.15–1.67)
*p*-value	<0.01		0.10	<0.01
Age at baseline adjusted HR (95% CI)	1.35 (1.07–1.70)	1.00 (referent)	1.05 (0.95–1.15)	1.30 (1.08–1.57)
*p*-value	0.01		0.34	<0.01
Multivariate HR^a^ (95% CI)	1.17 (0.92–1.49)	1.00 (referent)	1.09 (0.99–1.20)	1.33 (1.08–1.62)
*p*-value	0.21		0.09	<0.01
Cancer				
No. of deaths	22	381	178	35
Crude HR (95% CI)	1.66 (1.08–2.55)	1.00 (referent)	1.11 (0.93–1.33)	1.43 (1.01–2.02)
*p*-value	0.02		0.25	0.04
Age at baseline adjusted HR (95% CI)	1.50 (0.98–2.31)	1.00 (referent)	1.08 (0.90–1.29)	1.37 (0.97–1.94)
*p*-value	0.06		0.42	0.07
Multivariate HR^a^ (95% CI)	1.31 (0.83–2.06)	1.00 (referent)	1.12 (0.93–1.34)	1.45 (1.00–2.10)
*p*-value	0.25		0.25	0.051
CVD				
No. of deaths	27	468	195	38
Crude HR (95% CI)	1.66 (1.13–2.45)	1.00 (referent)	0.99 (0.84–1.17)	1.26 (0.91–1.76)
*p*-value	0.01		0.90	0.17
Age at baseline adjusted HR (95% CI)	1.32 (0.89–1.94)	1.00 (referent)	0.96 (0.81–1.14)	1.19 (0.85–1.65)
*p*-value	0.17		0.64	0.31
Multivariate HR^a^ (95% CI)	1.11 (0.74–1.67)	1.00 (referent)	0.97 (0.81–1.16)	1.13 (0.77–1.62)
*p*-value	0.62		0.72	0.51
Other				
No. of deaths	27	503	244	47
Crude HR (95% CI)	1.55 (1.05–2.28)	1.00 (referent)	1.15 (0.99–1.34)	1.46 (1.08–1.97)
*p*-value	0.03		0.07	0.01
Age at baseline adjusted HR (95% CI)	1.25 (0.85–1.83)	1.00 (referent)	1.11 (0.96–1.30)	1.37 (1.01–1.84)
*p*-value	0.26		0.16	0.04
Multivariate HR^a^ (95% CI)	1.11 (0.74–1.65)	1.00 (referent)	1.20 (1.02–1.41)	1.43 (1.03–1.99)
*p*-value	0.61		0.03	0.03

Note. *CI* confidence interval, *CVD* cardiovascular disease, *HR* hazard ratio

^a^ The multivariate HR has been adjusted for age at baseline (continuous variable), number of births (1, 2, 3, or ≥4), use of oral contraceptives (yes or no), consumption of green vegetables, carrot or pumpkin, orange (for each food, ≤1–2 times per month, 1–2 times per week, 3–4 times per week, or almost every day), consumption of coffee (<1, 1–2, 3–4, or ≥5 cups per day), smoking status (never, former, or currently smoking), alcohol drinking (never, former, or currently drinking), body mass index at baseline in kg/m^2^ (<18.5, 18.5–24.9, or ≥25.0), increase in body weight since age 20 years (<10.0 or ≥10.0 kg), walking duration (<1 or ≥1 h per day), sleeping time in hours per day (continuous variable), defecation frequency (≥1 time per day, 1 time per 2–3 days, 1 time per 4–5 days or ≤1 time per 6 days), years of education (<10, 10–12, or ≥13 years), ikigai (yes, uncertain, or no), perceived mental stress (high, moderate, or low), and history of diabetes (yes or no)

While no significant difference was apparent, there was an evident tendency toward a U-shaped relationship between age at first birth and the mortality because of cancer and cardiovascular diseases. The multivariate-adjusted HR for mortality because of cancer was 1.31 (95% CI, 0.83–2.06; *p* = .25) in the ≤19 years group and 1.45 (95% CI, 1.00–2.10; *p =* .051) in the ≥30 years group. For mortality because of cardiovascular disease, the corresponding figure was 1.11 (95% CI, 0.74–1.67; *p =* .62) in the ≤19 years group and 1.13 (95% CI, 0.77–1.62; *p =* .51) in the ≥30 years group. In addition, a U-shaped relationship was also observed between the age at first birth and mortality because of other causes. For the ≤19 years group, the HR was 1.11 (95% CI, 0.74–1.65; *p =* .61). A significantly elevated risk of 1.43 (95% CI, 1.03–1.99; *p =* .03) was observed in the ≥30 years group. The 25–29 years group also showed a significant increase in the HR of 1.20 (95% CI, 1.02–1.41; *p =* .03).

Table 3 indicates the associations between age at first birth and various causes of death. In terms of cancer mortality, that for gastric cancer was significantly increased in the ≤19 years group, the multivariate-adjusted HR being 2.82 (95% CI, 1.19–6.68; *p =* .02). While not reaching a significant level, increased mortality because of lung, colorectal, breast, and uterine cancers were indicated in the ≥30 years group. In terms of cardiovascular mortality, a U-shaped relationship was observed between age at first birth and mortality because of cerebral hemorrhage. The multivariate HR for cerebral hemorrhage mortality was 2.65 (95% CI, 1.12–6.25; *p =* .03) in the ≥30 years group, showing a significantly increased risk. Although not reaching a significant level, a tendency toward increased mortality because of stroke and cerebral infarction was indicated in the ≤19 years group. For other causes of mortality, a U-shaped relationship was observed between age at first birth and mortality because of pneumonia and other non-pneumonia diseases, although the differences did not reach a significant level.

**Table 3:** Cox Proportional Hazard Ratios (HRs) of Mortality Because of Selected Sites of Cancer and Subgroups of Cardiovascular Disease, the Ohsaki Cohort, Japan, 1995–2008

	Age at first birth (years)			
	≤19	20–24	25–29	≥30
	(*n* = 498)	(*n* = 13,607)	(*n* = 5,625)	(*n* = 894)
Cancer				
Gastric cancer				
No. of deaths	7	57	20	3
Multivariate HR^a^ (95% CI)	2.82 (1.19–6.68)	1.00 (referent)	0.93 (0.55–1.56)	0.8 (0.23–2.75)
*p*-value	0.02		0.77	0.72
Lung cancer				
No. of deaths	1	43	21	6
Multivariate HR^a^ (95% CI)	0.54 (0.07–3.99)	1.00 (referent)	1.09 (0.63–1.87)	2.17 (0.86–5.47)
*p*-value	0.55		0.76	0.10
Colorectal cancer				
No. of deaths	1	49	29	6
Multivariate HR^a^ (95% CI)	0.54 (0.07–3.95)	1.00 (referent)	1.36 (0.84–2.21)	1.65 (0.65–4.16)
*p*-value	0.54		0.22	0.29
Breast cancer				
No. of deaths	0	7	8	2
Multivariate HR^a^ (95% CI)	-	1.00 (referent)	3.28 (1.06–10.14)	5.78 (0.91–36.88)
*p*-value	1.00		0.04	0.06
Ovarian cancer				
No. of deaths	0	15	4	1
Multivariate HR^a^ (95% CI)	-	1.00 (referent)	0.53 (0.17–1.66)	1.31 (0.16–10.48)
*p*-value	1.00		0.28	0.80
Uterine cancer				
No. of deaths	0	10	4	1
Multivariate HR^a^ (95% CI)	-	1.00 (referent)	0.92 (0.27–3.12)	2.20 (0.23–21.37)
*p*-value	0.99		0.90	0.50
CVD				
Coronary heart disease				
No. of deaths	6	93	41	7
Multivariate HR^a^(95% CI)	1.07 (0.43–2.69)	1.00 (referent)	0.94 (0.64–1.38)	0.97 (0.43–2.18)
*p*-value	0.88		0.74	0.93
Stroke				
No. of deaths	15	235	93	19
Multivariate HR^a^(95% CI)	1.30 (0.77–2.22)	1.00 (referent)	0.92 (0.72–1.19)	1.24 (0.75–2.04)
*p*-value	0.33		0.53	0.40
Cerebral infarction				
No. of deaths	8	97	38	6
Multivariate HR^a^(95% CI)	1.45 (0.69–3.05)	1.00 (referent)	0.85 (0.57–1.27)	0.72 (0.30–1.72)
*p*-value	0.33		0.43	0.46
Cerebral hemorrhage				
No. of deaths	3	46	25	7
Multivariate HR^a^(95% CI)	1.45 (0.44–4.75)	1.00 (referent)	1.39 (0.84–2.31)	2.65 (1.12–6.25)
*p*-value	0.54		0.20	0.03
Subarachnoid hemorrhage				
No. of deaths	1	62	21	3
Multivariate HR^a^(95% CI)	0.39 (0.05–2.86)	1.00 (referent)	0.89 (0.53–1.49)	1.15 (0.35–3.80)
*p*-value	0.36		0.65	0.82
Other				
Pneumonia				
No. of deaths	7	92	51	10
Multivariate HR^a^(95% CI)	1.36 (0.58–3.15)	1.00 (referent)	1.41 (0.98–2.03)	1.87 (0.92–3.81)
*p*-value	0.48		0.06	0.09
External causes				
No. of deaths	2	28	16	2
Multivariate HR^a^(95% CI)	1.61 (0.37–6.94)	1.00 (referent)	1.37 (0.71–2.65)	1.13 (0.25–5.21)
*p*-value	0.53		0.35	0.87

Note. *CI* confidence interval, *CVD* cardiovascular disease, *HR* hazard ratio

^a^ The multivariate HR has been adjusted for age at baseline (continuous variable), number of births (1, 2, 3, or ≥4), use of oral contraceptives (yes or no), consumption of green vegetables, carrot or pumpkin, orange (for each food, ≤1–2 times per month, 1–2 times per week, 3–4 times per week, or almost every day), consumption of coffee (<1, 1–2, 3–4, or ≥5 cups per day), smoking status (never, former, or currently smoking), alcohol drinking (never, former, or currently drinking), body mass index at baseline in kg/m^2^ (<18.5, 18.5–24.9, or ≥25.0), increase in body weight since age 20 years (<10.0 or ≥10.0 kg), walking duration (<1 or ≥1 h per day), sleeping time in hours per day (continuous variable), defecation frequency (≥1 time per day, 1 time per 2–3 days, 1 time per 4–5 days or ≤1 time per 6 days), years of education (<10, 10–12, or ≥13 years), ikigai (yes, uncertain, or no) perceived mental stress (high, moderate, or low), and history of diabetes (yes or no)

Table 4 summarizes the relationship between maternal age at first birth and all-cause mortality stratified by baseline age and birth year of the first child. A U-shaped relationship was seen for women aged ≥60 years at the baseline (i.e., those born before 1935). In this age group, the multivariate HR was 1.52 (95% CI, 1.22–1.89; *p* < .01) in the ≥30 years group, and 1.11 (95% CI, 1.00–1.24; *p* = .049) in the 25–29 years group, showing a significantly increased risk. Although not statistically significant, there was a tendency toward increased mortality in the ≤19 years group. However, no association was seen between maternal age at first birth and all-cause mortality among women aged 40–59 years at the baseline (i.e., those born in 1935 or after). A U-shaped relationship was observed when participants delivered their first children before 1960. In this group, the multivariate HR was 1.33 (95% CI, 1.03–1.72; *p* = .03) in the ≥30 years group, showing a significantly increased risk. Although not reaching a significant level, there was a tendency toward increased mortality in the ≤19 years group. The interaction of each p-value (baseline age: *p* < .33; the birth year of the first child: *p* < .58) was not statistically significant.

**Table 4:** Multivariate HR of Mortality According to Age at First Birth by Generation, the Ohsaki Cohort, Japan, 1995–2008

	Age at first birth (years)				
	≤19	20–24	25–29	≥30	
	(*n* = 498)	(*n* = 13,607)	(*n* = 5,625)	(*n* = 894)	*p*-interaction
Born year (Age at baseline)					
< 1935 (≥60)					0.33
No. of deaths	68	1169	528	104	
Multivariate HR^a^ (95% CI)	1.18 (0.91–1.52)	1.00 (referent)	1.11 (1.00–1.24)	1.52 (1.22–1.89)	
*p*-value	0.21		0.049	<0.01	
1935–1955 (40–59)					
No. of deaths	8	183	89	16	
Multivariate HR^a^ (95% CI)	1.01 (0.49–2.08)	1.00 (referent)	1.30 (1.00–1.69)	1.43 (0.83–2.46)	
*p*-value	0.99		0.051	0.20	
Birth year of the first child					
< 1960					0.58
No. of deaths	73	1203	486	72	
Multivariate HR^a^ (95% CI)	1.18 (0.92–1.51)	1.00 (referent)	1.05 (0.94–1.17)	1.33 (1.03–1.72)	
*p*-value	0.19		0.38	0.03	
1960-					
No. of deaths	3	149	131	48	
Multivariate HR^a^ (95% CI)	0.61 (0.19–1.94)	1.00 (referent)	1.26 (0.98–1.63)	1.45 (0.97–2.16)	
*p*-value	0.40		0.07	0.07	

Note. *CI* confidence interval, *CVD* cardiovascular disease, *HR* hazard ratio

^a^ The multivariate HR has been adjusted for age at baseline (continuous variable), number of births (1, 2, 3, or ≥4), use of oral contraceptives (yes or no), consumption of green vegetables, carrot or pumpkin, orange (for each food, ≤1–2 times per month, 1–2 times per week, 3–4 times per week, or almost every day), consumption of coffee (<1, 1–2, 3–4, or ≥5 cups per day), smoking status (never, former, or currently smoking), alcohol drinking (never, former, or currently drinking), body mass index at baseline in kg/m^2^ (<18.5, 18.5–24.9, or ≥25.0), increase in body weight since age 20 years (<10.0 or ≥10.0 kg), walking duration (<1 or ≥1 h per day), sleeping time in hours per day (continuous variable), defecation frequency (≥1 time per day, 1 time per 2–3 days, 1 time per 4–5 days or ≤1 time per 6 days), years of education (<10, 10–12, or ≥13 years), ikigai (yes, uncertain, or no), perceived mental stress (high, moderate, or low), and history of diabetes (yes or no)

## Discussion

To investigate the effects of maternal age at first birth on the long-term mortality in mothers, we conducted a 14-year follow-up of 20,624 parous Japanese women aged between 40 and 79 years. Our results revealed a significantly increased all-cause mortality in the ≥30 years age group. Likewise, the ≤19 years group also showed a tendency towards increased all-cause mortalirty. The same U-shaped relationship was also demonstrated for cancer, cardiovascular diseases, and other diseases.

Previous studies have reported associations between all-cause mortality and age at first birth in both older and younger mothers. Increased all-cause mortality has been reported for African–American women with an age at first birth of ≥25 years [25, 26]. No similar studies have been conducted for women of other races. An elevated all-cause mortality has been found among women of several races with an age at first birth of ≤19 years [4, 23, 24]. Consistent with these other studies, our study found a increased mortality in both the ≤19 and ≥30 years groups.

However, no previous studies have demonstrated a U-shaped relationship. While other studies divided their subjects into two groups at a threshold age of either 20 or 25 years [22–24, 26], our study analyzed four age groups: ≤19 years, 20–24 years, 25–29 years, and ≥30 years. This quartile analysis indicated for the first time a U-shaped relationship between maternal age at first birth and mortality. Therefore, while some results of our study are consistent with those of others, this is the first report to demonstrate a U-shaped relationship where mortality increases when maternal age at first birth is either high (late childbearing) or low (early childbearing).

Few studies have examined associations between maternal age at first birth and cause-specific mortality. Henretta [24] demonstrates that the mortality because of cancer and heart and lung diseases was increased among women who had been <20 years old at the time of their first parturition. Our results demonstrated a U-shaped relationship for all-cause mortality as well as mortality because of cancer, cardiovascular diseases, and other causes. Previous studies agree with a positive correlation between maternal age at first birth and breast cancer risk [8–10]. Similarly, although significant differences were not detected becaus eof the small number of deaths, our study revealed a marked increase in breast cancer mortality in the ≥30 years group. For gastric cancer, our study demonstrated a significantly increased risk for women with a lower age at first birth. However, a previous study in western countries have indicated no association [19]. With regard to cardiovascular diseases, previous studies have reported an increased risk of stroke incidence [20] and a decreased risk of cardiovascular incidence [21] in women who were older at the time of their first parturition. In the present study, the mortality because of stroke was increased in women who had both early and late first births. By contrast, age at first birth revealed no association with the mortality because of heart disease.

Factors responsible for excess risk among mothers of early childbearing have been discussed. First, socio-economic status was lower among mothers who had their first births before 20 years of age as compared with mothers 20 years or older [28]. Second, Grundy and Kravdal [23] have pointed out that burden of childbearing tend to be worse for women who become mothers at an early age, as such women often react to stressors by engaging in unhealthy behavior such as smoking and drinking. Third, one study [29] has reported that women who become pregnant at an early age may experience difficulty losing the weight gained during pregnancy and returning to their pre-pregnancy weight. This means that a tendency for obesity may continue thus playing a long-term role in increasing the risk of cardiovascular diseases. In our study, the abovementioned variables were considered as covariates (such as years of education, perceived mental stress, smoking status, alcohol drinking, BMI and increase in body weight since age 20 years). The results revealed a increased mortality among mothers of early childbearing. The association between maternal age at first birth and mortality was independent of these covariates. Therefore, other mechanism may be related than these variables.

On the other hand, the factors for excess risk among mothers of late childbearing is poorly understood. The only exception is breast cancer where some women who did not have a full-term pregnancy until age 30 may already have had cells that had undergone early stages of malignant transformation, and pregnancy could have stimulated the growth of these mutated cells (Gao et al., [30]).

Regarding the risk of late childbearing, we need to consider self-selection bias of women who were older at the time of their first birth. Women who were ≥65 years old at the time of our baseline survey in 1994 had been born before 1929 and were in their 20s between 1950 and 1960. At that time in Japan, the mean age at first marriage was between 23.0 and 24.4 years [31] and the mean age at first birth was between 24.4 and 25.4 years [32]. Furthermore, the proportion of unmarried women aged between 30 and 34 years was only 5.7–9.4% [33]. During that period, women giving birth for the first time after 30 years of age would have accounted for only a very small minority. The timing of marriage, pregnancy, and childbirth may have been delayed because of physical weakness or Japanese women having a job at that time had a tendency to marry late. These biases may have contributed to the increased long-term mortality among women who were older at the time of their first birth. Today, however, women have more career opportunities and the effects of this bias appear to be diminishing. Moreover, when comparing two groups of maternal ages at first birth, the ≥30 years and 20–24 years groups, the proportion of participants with university or higher degrees was higher in the ≥30 years group. In general, it is thought that social and economical privileges will provide positive influences on health, but our study showed that regardless of educational status, the mortality was high in the ≥30 years group when compared to the 20–24 years group. Therefore, long-term mortality may be more strongly affected by maternal age at first birth than by educational status. Age-stratified analysis demonstrated a U-shaped relationship between maternal age at first birth and all-cause mortality only in the ≥60 years age group at the baseline (women born before 1935). On the other hand, this pattern was not observed among women who were <60 years old at the baseline (born in 1935 or later). This may reflect a weakening of the self-selection bias mentioned above. However, as mortality was notably low for individuals <60 years old, the effect may have been simply because of a lack of statistical power for detecting differences in risk among the four groups divided by maternal age at first birth. Accordingly, it is premature to conclude whether the higher risk of being older at the time of first birth is declining among younger women. Furthermore, the same result was obtained from the birth year of the first child. In the birth year of the first child, the participants who delivered their first children before 1960 showed a U-shaped relationship, but the same tendency was not observed in those who delivered their first children after 1960. Because this result may have been simply due to a lack of statistical power, just like with the baseline age, the conclusion should be carefully interpreted. The interaction of each baseline age and the birth year of the first child was not statistically significant. Childbirth appears to be strongly affected by historical factors, such as social recognition for childbirth, including for the mothers, which varies depending on the historical time, and a woman’s educational status or a change in working situation, which affects maternal age at childbirth. However, our study did not show a statistically significant interaction of each baseline age and the birth year of the first child. From this result, maternal age at first birth may have had a great impact on long-term mortality in our study, even though we considered historical background at childbirth.

We described the significance of our study from the perspective of clinical aspects and social aspects. In terms of clinical aspects, our study indicated that, once again, maternal age at first birth is particularly important clinical and basic information for lifelong disease prevention in women, as well as for health maintenance and enhancement. In either advanced or young age for pregnancy, and in either advanced or young maternal age at first birth, adequate support and consideration have been offered in clinical practice. This is because, for example, advanced age increases the risks of maternal and fetal complications. In cases of young age, problems are likely to get worse due to a series of situations such as unmarried status, economic issues, and immature parenting, in addition to the risks mentioned above. These improvements have been mainly made by care and support during the perinatal period. However, attention towards these improvements has not occurred unless there was a particular incident at other times. Our study showed that maternal age at first birth was related to the long-term health condition of mothers; therefore, strengthening the prevention of diseases by health examinations and guidance, not only during the perinatal period but also throughout her life, will be necessary for a woman whose maternal age at first birth is ≥30 years or ≤19 years. In 2010, under the third basic plan for gender equality, the Japanese government set a goal to promote lifelong health support based on the characteristics of women who are likely to become pregnant and give birth [34]. Our study suggests that these efforts can be supported by epidemiologic evidence. In terms of social aspects, we suggest that our study could help a woman design her own personal life plan and organize her social environment. In Japan, although the average desired number of children among unmarried women has tended to gradually decline in recent years, this number increased from 2.03 in 2002 to 2.12 in 2010 [35]. Moreover, although the total fertility rate (TFR), which is the total number of births per woman, increased from 1.26 in 2005 to 1.39 in 2011 [36]. Whether or not a woman wants to have a baby and when/how many babies she wants, we think that judgement must be based on multiple factors, including her social, economic and physical conditions. In this study, we demonstrated that the timing of first birth is related to long-term health conditions; therefore, our findings can be useful for making a decision based on epidemiologic evidence on how each woman lives a healthy life during pregnancy and childbirth, and after birth. In order to achieve a healthy life, a healthy social environment will be required, as will a woman’s self-awareness and judgement. Our findings can be a part of the basic information used to help organize and improve the social environment for pregnancy and childbirth, and can also contribute to lifelong health. The rising trend of the mean maternal age at first birth can be seen on a worldwide basis. Japan is one of the highest country of the average age at first birth. Therefore, the findings of this study can be considered a valuable knowledge for many other countries. Whether this trend poses a risk to the long-term health and longevity of mothers is a matter of serious public health concern and warrants continued monitoring.

The present study had several strengths. First, the participants were female members of the general public living in a local community. Consequently, the study findings can be generalized to women in other communities in Japan. In addition, the baseline response rate was high at 95.2% and thus was a very significant study strength. Second, our study cohort of 20,624 participants was very large and followed up for 14 years. Furthermore, we were able to confirm all deaths and their causes, excluding only those of individuals who had moved outside the catchment area. These two attributes suggest the high validity of our results.

Our study had some limitations. First, with regard to an association between maternal age at first birth and mortality, although we had the large number of deaths studied so far, the results of the associations between age at first birth and various causes of death might have been because of chance. Second, in this study we obtained maternal age at first birth by questioning “How old were you when you first gave birth (including stillbirths)?” Because this question did not imply a miscarriage or abortion that women may have had previously, we could not obtain data associated with these situations. As described earlier, if the timing of first birth affects long-term health conditions, the time of miscarriages or abortions will also be an important source of information. We need a further study including these basic facts in order to investigate the effects of time of miscarriages and abortions on long-term health. Third, this study was conducted in only one local city, and may have been influenced by characteristics of the specific region; therefore, we must be careful in generalizing study results. Future studies will need to expand the investigation into several other geographic areas. Finally, because the baseline information was obtained by self-administered questionnaires, some degree of bias may have been unavoidable.

## Conclusions

This study has demonstrated an increased long-term mortality in two groups of mothers: those who gave birth at ≤19 and ≥30 years of age. Maternal age at first birth has been rising globally. Whether or not this trend poses a risk to the long-term health and longevity of mothers is a serious public health concern, and continued monitoring of this issue is warranted.

## Appendix

**Table 5:** Cox Proportional Hazard Ratios (HRs) of Mortality Because of Cardiovascular Disease and Cancer According to Age at First Birth, Except for Deaths of Less than 1 Year After Follow-up, the Ohsaki Cohort, Japan, 1995–2008

	Age at first birth (years)			
	≤19	20–24	25–29	≥30
	(*n* = 492)	(*n* = 13,551)	(*n* = 5,589)	(*n* = 891)
Person-years	5,345	153,218	64,423	9,880
All-cause				
No. of deaths	70	1,296	581	117
Crude HR (95% CI)	1.56 (1.23–1.98)	1.00 (referent)	1.06 (0.96–1.17)	1.41 (1.16–1.70)
*p*-value	<0.01		0.22	<0.01
Age at baseline adjusted HR (95% CI)	1.30 (1.02–1.66)	1.00 (referent)	1.03 (0.93–1.13)	1.33 (1.10–1.60)
*p*-value	0.03		0.59	<0.01
Multivariate HR^a^ (95% CI)	1.12 (0.87–1.44)	1.00 (referent)	1.07 (0.96–1.18)	1.35 (1.10–1.66)
*p*-value	0.39		0.21	<0.01
Cancer				
No. of deaths	19	363	167	35
Crude HR (95% CI)	1.51 (0.95–2.39)	1.00 (referent)	1.09 (0.91–1.31)	1.50 (1.06–2.12)
*p*-value	0.08		0.34	0.02
Age at baseline adjusted HR (95% CI)	1.37 (0.86–2.18)	1.00 (referent)	1.06 (0.88–1.27)	1.44 (1.02–2.04)
*p*-value	0.18		0.55	0.04
Multivariate HR^a^ (95% CI)	1.17 (0.71–1.90)	1.00 (referent)	1.09 (0.90–1.32)	1.50 (1.04–2.18)
*p*-value	0.54		0.37	0.03
CVD				
No. of deaths	24	444	178	36
Crude HR (95% CI)	1.56 (1.04–2.35)	1.00 (referent)	0.95 (0.80–1.13)	1.26 (0.90–1.77)
*p*-value	0.03		0.57	0.18
Age at baseline adjusted HR (95% CI)	1.24 (0.82–1.87)	1.00 (referent)	0.92 (0.78–1.10)	1.19 (0.84–1.67)
*p*-value	0.30		0.37	0.33
Multivariate HR^a^ (95% CI)	1.03 (0.67–1.59)	1.00 (referent)	0.92 (0.77–1.11)	1.10 (0.76–1.60)
*p*-value	0.39		0.61	0.69
Other				
No. of deaths	27	489	236	46
Crude HR (95% CI)	1.60 (1.08–2.35)	1.00 (referent)	1.14 (0.98–1.34)	1.47 (1.09–1.99)
*p*-value	0.02		0.09	0.01
Age at baseline adjusted HR (95% CI)	1.29 (0.87–1.90)	1.00 (referent)	1.11 (0.95–1.29)	1.38 (1.02–1.86)
*p*-value	0.20		0.20	0.04
Multivariate HR^a^ (95% CI)	1.14 (0.76–1.70)	1.00 (referent)	1.20 (1.02–1.41)	1.49 (1.07–2.07)
*p*-value	0.52		0.03	0.02

Note. *CI* confidence interval, *CVD* cardiovascular disease, *HR* hazard ratio

^a^ The multivariate HR has been adjusted for age at baseline (continuous variable), number of births (1, 2, 3, or ≥4), use of oral contraceptives (yes or no), consumption of green vegetables, carrot or pumpkin, orange (for each food, ≤1–2 times per month, 1–2 times per week, 3–4 times per week, or almost every day), consumption of coffee (<1, 1–2, 3–4, or ≥5 cups per day), smoking status (never, former, or currently smoking), alcohol drinking (never, former, or currently drinking), body mass index at baseline in kg/m^2^ (<18.5, 18.5–24.9, or ≥25.0), increase in body weight since age 20 years (<10.0 or ≥10.0 kg), walking duration (<1 or ≥1 h per day), sleeping time in hours per day (continuous variable), defecation frequency (≥1 time per day, 1 time per 2–3 days, 1 time per 4–5 days or ≤1 time per 6 days) years of education (<10, 10–12, or ≥13 years), ikigai (yes, uncertain, or no), perceived mental stress (high, moderate, or low), and history of diabetes (yes or no)

## Abbreviations

BMI: Body mass index
CI: Confidence interval
CVD: Cardiovascular disease
HR: Hazard ratio
ICD: International classification of disease
NHI: National health insurance
OECD: Organisation for economic co-operation and development
SD: Standard deviation
TFR: Total fertility rate
